# Evolution and function of neurocognitive systems in non-human animals

**DOI:** 10.1038/s41598-021-02736-8

**Published:** 2021-12-08

**Authors:** Elisa Frasnelli

**Affiliations:** 1 CIMeC Center for Mind/Brain Sciences, University of Trento, Piazza della Manifattura 1, 38068 Rovereto, TN Italy; 2 School of Life Sciences, University of Lincoln, Lincoln, Lincolnshire, LN6 7DL UK

**Keywords:** Neuroscience, Physiology, Evolution, Genetics, Zoology

## Abstract

Advances in cognitive neuroscience and neurotechnology have increased our understanding of the neurobiological mechanisms underlying cognitive processes. This Collection brings together research in animal behaviour and cognition, with studies investigating their physiology, neural mechanisms, and genetic bases, in order to provide insight into the function and evolution of neurocognitive systems.

The variety and richness of organisms found in nature offers scientists plenty of opportunities to investigate the many facets of biological complexity, and formulate novel research questions and hypotheses. This does not only apply to species-specific characteristics but, even more interestingly, to those features that can be observed across the animal kingdom. Comparative studies in psychology and neuroscience have proved to be an effective tool in advancing our understanding of the mechanisms underlying specific cognitive abilities, as well as their development, function, and evolution. This Collection gathers more than 40 contributions by scientists from all over the world, addressing different questions, in a variety of invertebrate and vertebrate species. The research comprises behavioural, electrophysiological, molecular, genetic, and neuroimaging studies, with the common aim of contributing to our understanding of the evolution and function of cognition.

Both the similarity and differences among species can help us understand the evolution of neurocognitive features and behaviours. Species as close to humans as primates enable scientists to look at brain structures and pathways (e.g., sulcal morphology^1^; fast visual responses in the amygdala^2^) and behavioural characteristics thought to be exclusive to our species (e.g., left-cradling bias^3^; lateral eye bias^4^; handedness^5^; abstract concept learning^6^), and investigate their neuroanatomical, genetic, and environmental basis (e.g., mutual gaze in social communication^7^). At the same time, the study and comparison of phylogenetically distant species, like birds or fish, can be ideal for testing predictions about the generality and conservation of brain mechanisms across the evolutionary tree (e.g., inhibition of return in barn owls^8^; Approximate Number System in zebrafish^9^; numerical discrimination in domestic chicks^10^; visual mental manipulation in Grey parrot, children, and human adults^11^). On the other hand, comparing related species may also reveal species-specific differences, that highlight the evolutionary divergence of neuronal circuit functions (e.g., the synaptic plasticity and the key molecules regulating it in shrews, mice, and bats^12^).

In some of the studies included in this Collection, the same tasks are presented to different species, revealing interesting inter-specific differences in the behavioural response (e.g., in re-orientation spatial skills in different species of fish^13^; in the use of geometric cues by rats and chicks^14^). Relatedly, comparative research of this sort sometimes raises questions about the conditions in which different species are tested. Some of the papers thus investigate potential methodological limitations in the study of animal behaviour. For instance, Morandi-Raikova and Mayer^15^ show that experimental manipulation can affect the behaviour and the neural activity of the tested animal. It is also essential to provide replication of previous findings, either to substantiate the claims presented, or—as done by Lemaire^16^ in this Collection—to question them.

Behavioural studies are extremely valuable in testing theories of adaptation (e.g., natural pedagogy in dogs^17^), emotional processing (e.g., in dogs^18^), rationality (e.g., in mice^19^), decision-making (e.g., in pigeons^20^), learning (e.g., in bees^21^), and memory (e.g., in cuttlefish^22^; in mice^23^), but they can also reveal intriguing possible relationships, such as between behaviour, brain mass, and lifespan, as shown by Kaplan^24^ in Australian native birds. Animals raised in laboratories give scientists the possibility to study specific cognitive abilities in naïve individuals (e.g., the use of sensory cues in social learning in naïve gerbils^25^) and allow scientists to control for experience, especially in early life (e.g., the effect of early life stress on memory formation in the nematode *C. elegans*^26^; the effect of long-lasting social isolation and re-socialization on cognitive performance and brain activity in *Octodon degus*^27^). This is particularly valuable in precocial species such as chicken, which are ideal to investigate in-born predispositions to attend to specific stimuli (e.g.,^28^), and to exploit imprinting procedures to elucidate the ecological function of statistical learning (e.g.,^29^). Free living animals, on the other hand, allow scientists to study how species behave in their natural environment. Louder et al.^30^ investigated behavioural plasticity and gene expression in response to different antagonistic stimuli in free-living red-winged blackbirds and found shared molecular and behavioural pathways involved in the recognition of—and reaction to—both evolutionarily old and new enemies. Wild-caught and laboratory individuals of the same species can also be tested to compare the possible group-specific performances in cognitive tasks, as shown in the study by Rössler and colleagues^31^, who investigated the ability to innovate in Goffin’s cockatoos. Furthermore, through the study of groups of animals of the same species living in different habitats, it is possible to assess the effect of the environment on behaviour (e.g., the behavioural adjustment of striped field mice to human disturbance^32^).

Notably, behavioural studies can be combined with molecular, electrophysiological, neuro-imaging, and genetic techniques to pinpoint the neural mechanisms underpinning behaviour (e.g., immediate early gene expression of multi-component behaviour in pigeons^33^; protein products of the immediate early genes in response to exposure to conspecific contact calls in male budgerigars^34^; electrophysiological recordings of neuronal activity during song broadcast and social relationships in starlings^35^; cortical activity and motor behaviour to establish levels of arousal in rodents^36^). Furthermore, they can be used to investigate the effects of a treatment on behaviour (e.g., chronic consumption of D-amino acids on spatial learning and expression of NMDA receptors in mice^37^) and on their neural signature (e.g., anxiolytic high-frequency electrical stimulation of the bed nucleus of the stria terminalis in rats on c-Fos expression^38^). Some species can be manipulated so that they express a marker such as GFP, in specific subpopulations of neurons, in order to study their differentiation and development (e.g., sex-differences in hippocampal neurons in mice^39^); whereas in other species, genetic lines in which a particular gene is silenced can be produced, which is invaluable for providing insights into the genetic substrates of specific neurobiological features or behaviours (e.g., lateralization in *Drosophila*^40^; impulsivity in rats^41^).

The papers published in this Collection highlight how fundamental studies on animal models are to our understanding of the functioning, development, and evolution of neurocognitive systems, and teach us, through the comparative approach, that we share more than we think with even the more evolutionarily distant species.

## References

[1] Miller JA, Voorhies WI, Li X (2020). Sulcal morphology of ventral temporal cortex is shared between humans and other hominoids. Sci. Rep..

[2] Cleeren E, Popivanov ID, Van Paesschen W (2020). Fast responses to images of animate and inanimate objects in the nonhuman primate amygdala. Sci. Rep..

[3] Boulinguez-Ambroise G, Pouydebat E, Disarbois É (2020). Human-like maternal left-cradling bias in monkeys is altered by social pressure. Sci. Rep..

[4] Donati G, Davis R, Forrester GS (2020). Gaze behaviour to lateral face stimuli in infants who do and do not receive an ASD diagnosis. Sci. Rep..

[5] Forrester GS, Davis R, Malatesta G (2020). Evolutionary motor biases and cognition in children with and without autism. Sci. Rep..

[6] Rugani R, Platt ML, Chen Z (2020). Middle identification for rhesus monkeys is influenced by number but not extent. Sci. Rep..

[7] Hopkins WD, Mulholland MM, Reamer LA (2020). The role of early social rearing, neurological, and genetic factors on individual differences in mutual eye gaze among captive chimpanzees. Sci. Rep..

[8] Lev-Ari T, Zahar Y, Agarwal A (2020). Behavioral and neuronal study of inhibition of return in barn owls. Sci. Rep..

[9] Messina A, Potrich D, Schiona I (2020). Response to change in the number of visual stimuli in zebrafish: A behavioural and molecular study. Sci. Rep..

[10] Rugani R, Loconsole M, Simion F (2020). Individually distinctive features facilitate numerical discrimination of sets of objects in domestic chicks. Sci. Rep..

[11] Pailian H, Carey SE, Halberda J (2020). Age and species comparisons of visual mental manipulation ability as evidence for its development and evolution. Sci. Rep..

[12] Beed P, Ray S, Velasquez LM (2020). Species-specific differences in synaptic transmission and plasticity. Sci. Rep..

[13] Sovrano VA, Baratti G, Potrich D (2020). The geometry as an eyed fish feels it in spontaneous and rewarded spatial reorientation tasks. Sci. Rep..

[14] Lee SA, Austen JM, Sovrano VA (2020). Distinct and combined responses to environmental geometry and features in a working-memory reorientation task in rats and chicks. Sci. Rep..

[15] Morandi-Raikova A, Mayer U (2020). The effect of monocular occlusion on hippocampal c-Fos expression in domestic chicks (G*allus gallus)*. Sci. Rep..

[16] Lemaire BS (2020). No evidence of spontaneous preference for slowly moving objects in visually naïve chicks. Sci. Rep..

[17] Neilands P, Kingsley-Smith O, Taylor AH (2021). Dogs’ insensitivity to scaffolding behaviour in an A-not-B task provides support for the theory of natural pedagogy. Sci. Rep..

[18] Bolló H, Kovács K, Lefter R (2020). REM versus non-REM sleep disturbance specifically affects inter-specific emotion processing in family dogs (C*anis familiaris)*. Sci. Rep..

[19] Schneider NA, Ballintyn B, Katz D (2021). Parametric shift from rational to irrational decisions in mice. Sci. Rep..

[20] Manns M, Otto T, Salm L (2021). Pigeons show how meta-control enables decision-making in an ambiguous world. Sci. Rep..

[21] Aguiar JMRBV, Giurfa M, Sazima M (2020). A cognitive analysis of deceptive pollination: Associative mechanisms underlying pollinators’ choices in non-rewarding colour polymorphic scenarios. Sci. Rep..

[22] Billard P, Clayton NS, Jozet-Alves C (2020). Cuttlefish retrieve whether they smelt or saw a previously encountered item. Sci. Rep..

[23] Cruz-Sanchez A, Dematagoda S, Ahmed R (2020). Developmental onset distinguishes three types of spontaneous recognition memory in mice. Sci. Rep..

[24] Kaplan G (2020). Play behaviour, not tool using, relates to brain mass in a sample of birds. Sci. Rep..

[25] Paraouty N, Charbonneau JA, Sanes DH (2020). Social learning exploits the available auditory or visual cues. Sci. Rep..

[26] Gecse E, Gilányi B, Csaba M (2019). A cellular defense memory imprinted by early life toxic stress. Sci. Rep..

[27] Rivera DS, Lindsay CB, Oliva CA (2020). Effects of long-lasting social isolation and re-socialization on cognitive performance and brain activity: A longitudinal study in *Octodon degus*. Sci. Rep..

[28] Versace E, Ragusa M, Vallortigara G (2019). A transient time window for early predispositions in newborn chicks. Sci. Rep..

[29] Santolin C, Rosa-Salva O, Lemaire BS (2020). Statistical learning in domestic chicks is modulated by strain and sex. Sci. Rep..

[30] Louder MIM, Lafayette M, Louder AA (2020). Shared transcriptional responses to con- and heterospecific behavioral antagonists in a wild songbird. Sci. Rep..

[31] Rössler T, Mioduszewska B, O’Hara M (2020). Using an Innovation Arena to compare wild-caught and laboratory Goffin’s cockatoos. Sci. Rep..

[32] Dammhahn M, Mazza V, Schirmer A (2020). Of city and village mice: Behavioural adjustments of striped field mice to urban environments. Sci. Rep..

[33] Rook N, Letzner S, Packheiser J (2020). Immediate early gene fingerprints of multi-component behaviour. Sci. Rep..

[34] Satoh R, Eda-Fujiwara H, Watanabe A (2021). Memory-specific correlated neuronal activity in higher-order auditory regions of a parrot. Sci. Rep..

[35] Cousillas H, Henry L, George I (2020). Lateralization of social signal brain processing correlates with the degree of social integration in a songbird. Sci. Rep..

[36] Gao S, Calderon DP (2020). Robust alternative to the righting reflex to assess arousal in rodents. Sci. Rep..

[37] Zachar G, Kemecsei R, Papp SM (2021). D-Aspartate consumption selectively promotes intermediate-term spatial memory and the expression of hippocampal NMDA receptor subunits. Sci. Rep..

[38] Luyck K, Scheyltjens I, Nuttin B (2020). c-Fos expression following context conditioning and deep brain stimulation in the bed nucleus of the stria terminalis in rats. Sci. Rep..

[39] Brandt N, Löffler T, Fester L (2020). Sex-specific features of spine densities in the hippocampus. Sci. Rep..

[40] Versace E, Caffini M, Werkhoven Z (2020). Individual, but not population asymmetries, are modulated by social environment and genotype in *Drosophila melanogaster*. Sci. Rep..

[41] Jupp B, Pitzoi S, Petretto E (2020). Impulsivity is a heritable trait in rodents and associated with a novel quantitative trait locus on chromosome 1. Sci. Rep..

